# From Real-World Practice to an Ideal Rehabilitation Pathway in Osteoarthritis: A Delphi Consensus on Patient Itineraries

**DOI:** 10.3390/jcm15083047

**Published:** 2026-04-16

**Authors:** Helena Bascuñana-Ambrós, Alex Trejo-Omeñaca, Carlos Cordero-García, Sergio Fuertes-González, Juan Ignacio Castillo-Martín, Michelle Catta-Preta, Jan Ferrer-Picó, Josep Maria Monguet-Fierro, Jacobo Formigo-Couceiro

**Affiliations:** 1 Physical Medicine and Rehabilitation Department, Hospital de la Santa Creu i Sant Pau, Campus Salut, 08025 Barcelona, Spain; 2 Spanish Society of Physical Medicine and Rehabilitation (SERMEF), 28016 Madrid, Spain; carlos.cordero.sspa@juntadeandalucia.es (C.C.-G.); sfuertes@saludcastillayleon.es (S.F.-G.); juanignacio.castillo@salud.madrid.org (J.I.C.-M.); josep@innex.io (J.M.M.-F.); jacobo.formigo.couceiro@sergas.es (J.F.-C.); 3 Institut pel Futur, 17190 Salt, Spain; alex@innex.io (A.T.-O.); michelle@innex.io (M.C.-P.); jan@innex.io (J.F.-P.); 4 Innex Labs, 08800 Vilanova i la Geltrú, Spain; 5 Universitat Politècnica de Catalunya, ETSEIB, 08028 Barcelona, Spain; 6 Physical Medicine and Rehabilitation Department, Hospital Universitario Juan Ramón Jiménez, 21005 Huelva, Spain; 7 Physical Medicine and Rehabilitation Department, Hospital Santiago Apostol, 09200 Miranda de Ebro, Spain; 8 CRR, Grupo Recoletas Salud, 09004 Burgos, Spain; 9 Physical Medicine and Rehabilitation Department, Hospital Universitario 12 Octubre, 28041 Madrid, Spain; 10 Physical Medicine and Rehabilitation Department, A Coruña University Hospital, 15006 A Coruña, Spain; 11 Rehabilitation Research Group, Biomedical Research Institute of A Coruña (INIBIC), 15006 A Coruña, Spain

**Keywords:** knee osteoarthritis, physical medicine and rehabilitation, Delphi consensus, real-time Delphi, care pathway, rehabilitation itinerary

## Abstract

**Background**: Care for knee osteoarthritis (KOA) is frequently fragmented, and pathway-level decisions within Physical Medicine and Rehabilitation (PM&R) are influenced by local organizations. The objective of this study was to identify areas of agreement and disagreement among PM&R experts and to translate these into a clinically interpretable, function-oriented care pathway for knee osteoarthritis (KOA) within rehabilitation services. **Methods:** A two-round Real-Time Delphi study was conducted using the SmartDelphi web platform. A steering committee of five PM&R physicians developed a 37-item questionnaire covering referral/access, functional and outcome assessment, conservative management, escalation/referral thresholds, and follow-up/discharge. Round 1 was online (SERMEF osteoarthritis working group; 46 invited, 40 completed; 87.0%) with responses collected until 30 April 2025. Round 2 was an in-person, facilitated validation round on 30 May 2025 at the SERMEF Congress (A Coruña; 85 invited, 70 completed; 82.4%). Items were rated on a 6-point Likert scale; consensus strength was defined by interquartile range (IQR): strong (0–1) vs. weak (≥2). No patient-level data were collected; participant characteristics were comparable across rounds, suggesting consensus refinement reflected deliberation rather than panel shifts over time. **Results:** Consensus supported a longitudinal, function-first pathway that was structured into five phases: entry/referral to PM&R; comprehensive functional assessment using a minimum outcomes dataset (pain VAS/NRS, WOMAC function, quality-of-life scale); multimodal conservative rehabilitation combining exercise/physiotherapy, education/self-management support, and indicated oral/topical therapies; reassessment-guided escalation in non-responders, reserving interventional PM&R techniques, multidisciplinary musculoskeletal pain-unit management, or orthopedic evaluation for persistent pain and/or functional limitation; and longitudinal monitoring with defined discharge criteria. **Conclusions:** SERMEF PM&R experts converged on an implementation-oriented, outcomes-driven KOA itinerary centred on functioning, conservative multimodal care, structured reassessment, and explicit discharge planning.

## 1. Introduction

Knee osteoarthritis (KOA) is a leading cause of chronic pain, reduced mobility, and years lived with disability, with substantial societal impact through work disability and healthcare utilization. Recent Global Burden of Disease (GBD) analyses document a sustained increase in OA-related burden since 1990 and project further growth to 2050, driven largely by population ageing and demographic transitions [1]. Given its high prevalence and direct effect on walking capacity and participation, KOA is a key target for pathway-based, high-value care.

Across major clinical practice guidelines, KOA management is consistently anchored in non-pharmacological, person-centred interventions—therapeutic exercise, education and self-management support, weight management when indicated, and biomechanical/assistive strategies—while pharmacological and interventional approaches are positioned as adjuncts within a stepped-care model [2,3,4]. Despite this convergence, real-world delivery remains heterogeneous and frequently fragmented across levels of care and specialties, contributing to unwarranted variation in the timing of conservative care, referral thresholds, continuity of follow-up, and alignment with patient priorities. In response, health-system quality initiatives have promoted measurable standards and pathway-based models to reduce variability and ensure timely access to evidence-based conservative management before escalation to specialist referral and surgical consideration [5,6].

Physical Medicine and Rehabilitation (PM&R) is positioned to coordinate a functioning-oriented itinerary for KOA that integrates pain management with disability reduction through personalized exercise prescription, movement and biomechanical optimization, education, and shared goal setting. In routine practice, PM&R commonly interfaces primary care, rheumatology/orthopedics, physiotherapy, and community resources, and can operationalise minimum assessment datasets and longitudinal follow-up models focused on functioning outcomes. However, key pathway decisions—such as sequencing of interventions, criteria and timing for referral, minimum standards for access to structured rehabilitation, and follow-up intensity—are not always fully resolved by comparative evidence and are influenced by local service organization and practice patterns.

In this context, formal consensus methods can produce implementation-ready recommendations when evidence is incomplete at the care-model level. Real-time digital Delphi approaches enable iterative rating with immediate aggregated feedback, improving efficiency and potentially reducing attrition compared with traditional multi-round designs [7]. For the present project, we used a Real-Time Delphi approach implemented with the SmartDelphi tool (www.smartdelphi.com) (accessed on 2 March 2026) [8], a digitally adapted Delphi model designed to support online consensus-building in healthcare with rapid feedback and structured participation.

Therefore, the objective of this study was to (1) identify areas of consensus and disagreement among PM&R experts regarding key clinical and organizational decisions in KOA management, and (2) translate item-level consensus into a structured, function-oriented rehabilitation pathway applicable to real-world practice.

## 2. Materials and Methods

### 2.1. Study Design and Digital Platform

We conducted a two-round modified Real-Time Delphi consensus study to define a function-oriented care itinerary for patients with knee osteoarthritis (KOA) managed within Physical Medicine and Rehabilitation (PM&R) services. The process was implemented using the SmartDelphi web-based platform version 2025 (www.smartdelphi.com), which enables structured online participation with real-time aggregation of responses and visual feedback, allowing participants to review collective trends while preserving response anonymity [8]. Participants in the online round were able to review aggregated group responses in real time and could modify their ratings iteratively during the response period, consistent with Real-Time Delphi methodology. In contrast, the second round did not involve iterative re-rating over time but functioned as a structured, facilitated validation step using the same feedback logic.

This study combined an initial asynchronous digital round with a subsequent facilitated validation round conducted in person. This hybrid configuration reflects contemporary adaptations of Delphi methodology that integrate digital real-time feedback mechanisms and controlled panel expansion to enhance representativeness and external validation, while maintaining consistent eligibility criteria and identical questionnaire structure across rounds [7,8].

### 2.2. Questionnaire Development

The Delphi questionnaire was developed by a steering committee of five PM&R physicians, each with >10 years of experience in the conservative management of knee OA. The committee’s mandate was to define a function-oriented itinerary centred on pathway decision points aligned with functional assessment and longitudinal management. Through iterative meetings and item refinement, the committee finalized 37 items covering the main stages of the KOA itinerary: assessment, conservative management, escalation criteria, referral thresholds, and follow-up. Drafting was conducted between January and February 2025, followed by a technical review and proofreading during the first week of March 2025 to correct minor inconsistencies.

To enhance content validity and ensure comprehensive coverage of relevant clinical domains, questionnaire development followed a structured triangulation approach. The initial item pool was informed by a targeted review of major international clinical practice guidelines for knee osteoarthritis management, including EULAR, OARSI, and NICE recommendations, as well as by previously published care pathway models and rehabilitation-oriented outcome frameworks. Rather than conducting a formal systematic review, the steering committee performed a structured evidence-informed mapping exercise to identify key decision points across the KOA care trajectory, including referral patterns, functional assessment standards, therapeutic sequencing, escalation criteria, and follow-up strategies.

**Figure 1:**
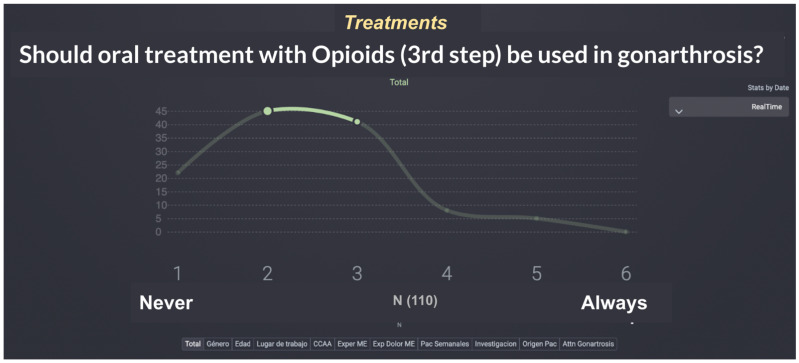
Sample screen of the SmartDelphi tool used to facilitate consensus in the Real-Time Delphi synchronous focus group.

Draft items were iteratively refined through multidisciplinary expert discussion to ensure clinical relevance, clarity, and coverage of the full rehabilitation pathway. Particular attention was paid to capturing both intervention-related and organizational decision nodes, reflecting the real-world coordination role of PM&R services. The final set of 37 items was considered to provide adequate representational coverage of the KOA rehabilitation itinerary while maintaining feasibility for participant completion and response quality, consistent with methodological recommendations for Delphi questionnaire design. Figure 1 shows a screenshot of how the feedback is presented to participants, showing descriptive statistics and options to check any stratification of the answers to the questionnaires.

### 2.3. Participants, Eligibility, and Recruitment

Eligible participants were PM&R physicians who were members of the Spanish Society of Physical Medicine and Rehabilitation (SERMEF) and had clinical experience in knee OA management. No formal sample size calculation was performed, consistent with Delphi methodology, which prioritizes expertise over statistical representativeness.

Round 1 (online, asynchronous). Participants were recruited from the SERMEF osteoarthritis working group. Invitations were distributed by SERMEF via email, and participants accessed the questionnaire through a web link sent to their email address. Round 1 responses were collected until 30 April 2025. The steering committee reviewed Round 1 outputs to evaluate item clarity and interpretability and to identify domains requiring consolidation prior to the in-person consensus exercise.

Round 2 (in-person, facilitated). Round 2 was conducted on 30 May 2025 during the SERMEF Congress in A Coruña. Participants joined by scanning a QR code displayed on the projection screen, providing direct access to the same online questionnaire.

A total of 46 eligible PM&R physicians from the SERMEF osteoarthritis working group were invited to participate in Round 1 of the Delphi process. Of these, 40 completed the questionnaire, corresponding to a response rate of 87.0%.

Round 2 was conducted during the SERMEF Congress and was designed as an expanded validation round, allowing participation from a broader group of clinicians with expertise in knee osteoarthritis management. A total of 85 physicians were invited to participate in this phase, of whom 70 completed the questionnaire, yielding a response rate of 82.4%.

Although Round 2 incorporated additional participants, the eligibility criteria and questionnaire structure remained identical across rounds, ensuring methodological consistency. The demographic and professional characteristics of participants were comparable across rounds, suggesting that consensus refinement primarily reflects iterative expert deliberation rather than shifts in panel composition.

To minimize recruitment bias, invitations were distributed centrally by the scientific society rather than by individual investigators. Participation was restricted to PM&R physicians with demonstrated clinical involvement in knee osteoarthritis management. The inclusion of a second round conducted during a national scientific congress increased panel heterogeneity and improved representativeness across different clinical settings and levels of experience.

As participation was voluntary, self-selection bias cannot be fully excluded. However, the high response rates and broad distribution of professional characteristics support the representativeness of the expert panel.

**Figure 2:**
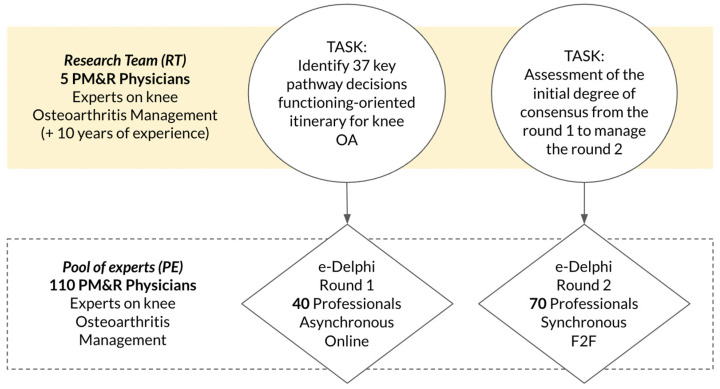
Tasks performed by the research team and participants in the two Delphi rounds.

Figure 2 summarizes the tasks performed by the research team and participants in the two Delphi rounds.

Rather than constituting two strictly sequential Delphi rounds with item modification, the process followed a hybrid real-time Delphi approach combining an initial asynchronous rating phase and a subsequent expanded validation round. The second round was designed to test the stability and external validity of the initial consensus in a broader expert sample, rather than to reconfigure the questionnaire.

The main difference between rounds was procedural: the first round was asynchronous, allowing extended interaction with the platform, whereas the second round was conducted synchronously in a facilitated setting. Eligibility criteria remained consistent across both rounds.

### 2.4. Delphi Procedure and Rating Scale

In both rounds, participants rated each item using a 6-point Likert scale (1 = strongly disagree; 6 = strongly agree). The digital platform enabled immediate aggregation and visualization of group responses to support structured consensus-building and facilitate interpretation during the in-person exercise.

### 2.5. Data Collection

In addition to item ratings, participants reported demographic and professional characteristics, including age, gender, geographic region, and years of clinical experience. No patient-level data were collected.

### 2.6. Consensus Criteria

Consensus strength was assessed using the interquartile range (IQR) of item ratings. Strong consensus was defined as IQR 0–1. Weak consensus was defined as IQR ≥ 2, indicating heterogeneous ratings and highlighting items requiring further consolidation and discussion in the subsequent consensus step.

No intermediate consensus category was predefined; therefore, narrative descriptions of agreement levels in the Results and Discussion are aligned strictly with these predefined thresholds.

### 2.7. Statistical Analysis

For each item and round, responses were summarized using the median and IQR. Items were classified according to the predefined consensus categories (strong vs. weak) based on IQR thresholds. Where relevant, the proportion of respondents selecting high-agreement categories (ratings 5–6) was also calculated to support the interpretability of agreement intensity. Analyses were performed with item-level denominators (i.e., *n* reported per item), and missing data were handled at the item level (pairwise).

Panel stability and representativeness were descriptively evaluated by comparing aggregated demographic and professional characteristics of participants across rounds.

Changes between rounds were interpreted as indicators of consensus stability and convergence rather than iterative item reformulation.

### 2.8. Ethical and Data Protection Considerations

Participation was voluntary. All participants received study information and provided electronic informed consent prior to questionnaire access. Data were collected and analyzed in anonymized form, and processing complied with applicable data-protection requirements (GDPR, EU 2016/679).

## 3. Results

### 3.1. Delphi Participants Rounds 1 and 2

The composition of the expert panel was comparable across rounds, although not identical. The second round incorporated additional participants recruited during the national congress, while maintaining the same eligibility criteria and questionnaire structure. Participants in both rounds shared comparable clinical and professional profiles, including years of experience in musculoskeletal medicine, active clinical practice, and institutional settings. No relevant differences were identified in terms of clinical profile, suggesting that changes in consensus primarily reflect the deliberative effect of the process rather than major shifts in participant characteristics. The characteristics of the SERMEF Physical Medicine and Rehabilitation (PM&R) physicians who participated in both Delphi rounds are summarized in Table 1.

### 3.2. Construction of the Function-Oriented KOA Rehabilitation Pathway

**Figure 3:**
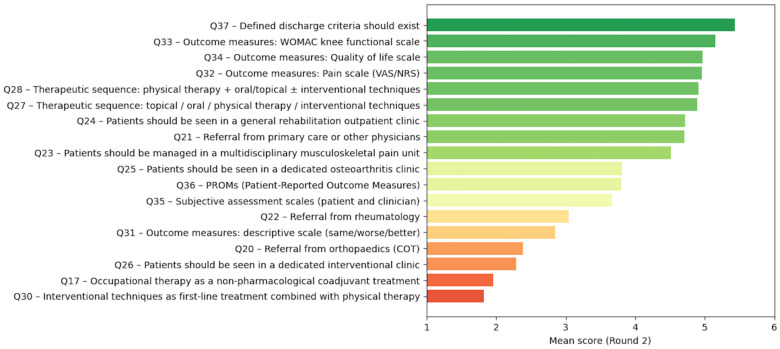
Mean item ratings across the two Delphi rounds (Round 1 vs. Round 2).

Beyond item-level agreement, the Delphi exercise supported the integration of consensus statements into a coherent, function-oriented rehabilitation pathway for patients with KOA managed within PM&R services. Using items that achieved strong consensus (IQR 0–1) and mapping their logical sequence within routine clinical workflows, we constructed a sequential model of care structured around key decision points and longitudinal management (Table 2; Figure 3).

The resulting pathway is organized into five interrelated clinical phases: (1) entry and referral, (2) comprehensive functional assessment, (3) structured conservative intervention, (4) escalation and interventional decision-making, and (5) longitudinal monitoring and discharge planning.

#### 3.2.1. Entry and Referral Phase

Consensus supported that KOA patients should typically access PM&R through referral from primary care or other physicians and be evaluated within a general rehabilitation outpatient setting (Table 2). The entry phase includes confirmation of the clinical diagnosis, exclusion of alternative causes of knee pain, and identification of factors requiring prioritization or early referral.

#### 3.2.2. Comprehensive Functional Assessment Phase

Strong consensus emerged for the systematic use of multidimensional outcome measures to guide clinical decision-making, including pain assessment scales (VAS/NRS), disease-specific functional measures such as WOMAC and other validated instruments used in clinical practice (e.g., KOOS), as well as quality-of-life scales (Table 2). These measures were incorporated as core components of baseline assessment and subsequent reassessment within the pathway. Items that did not reach strong consensus in Round 1 but were retained in the final pathway (e.g., WOMAC, pain scales) were incorporated based on their improved agreement in Round 2 and their role as widely used reference measures in clinical practice.

#### 3.2.3. Structured Conservative Intervention Phase

The panel supported a multimodal conservative management approach coordinated within PM&R services. Therapeutic exercise was the most consistently endorsed component (IQR 0), whereas other elements such as physiotherapy showed more heterogeneous agreement. In the final pathway, these components are integrated within a multimodal framework, reflecting their complementary role in clinical practice rather than an equivalent level of consensus support. Overall, the pathway combines exercise-based interventions with pharmacological and topical therapies when clinically indicated and considers interventional techniques as adjuncts rather than standalone strategies (Table 2).

#### 3.2.4. Escalation and Interventional Decision-Making Phase

Consistent with the lowest-rated items, experts did not support the use of interventional techniques as first-line treatment (Table 2). Interventions were positioned in the pathway as escalation options for patients with persistent pain and/or functional limitation despite adequate conservative management, with decision nodes informed by reassessment outcomes.

#### 3.2.5. Longitudinal Monitoring and Discharge Planning Phase

One of the highest-rated statements in the Delphi process was the need for defined discharge criteria (Table 2). The pathway, therefore, incorporates periodic follow-up and reassessment using the same validated measures applied at baseline to monitor response and guide continuation, optimization, escalation, or discharge decisions. Discharge is framed as a planned transition following achievement of predefined criteria and functional stabilization.

Taken together, these consensus-derived elements define a structured, function-oriented KOA rehabilitation pathway characterized by standardized assessment, multimodal conservative care as the foundation of management, escalation based on treatment response, and explicit discharge planning supported by repeated outcome measurement (Table 2; Figure 3).

### 3.3. Mapping of Delphi Consensus Statements to the Rehabilitation Pathway Model

To enhance methodological transparency, the construction of the rehabilitation pathway was based on a structured mapping process linking individual Delphi consensus statements to specific clinical phases and decision nodes within the proposed care model. Items achieving strong consensus (IQR 0–1) were considered core structural components of the pathway, whereas items showing moderate consensus informed supportive or contextual elements.

The mapping process followed three sequential steps. First, consensus items were grouped according to their primary clinical domain, including referral and access to care, functional assessment, therapeutic intervention, escalation criteria, and follow-up strategies. Second, items were organized into a chronological sequence reflecting routine clinical workflows in KOA management. Third, related items were synthesized into decision nodes defining transitions between pathway phases.

For example, items supporting referral from primary care and evaluation within general rehabilitation outpatient clinics were mapped to the entry phase of the pathway. Items demonstrating strong agreement regarding the use of validated outcome measures, including WOMAC, pain scales, and quality-of-life instruments, were incorporated into the comprehensive functional assessment phase. Statements supporting multimodal conservative management informed the intervention phase, whereas items rejecting interventional procedures as first-line treatment contributed to defining escalation criteria. Finally, consensus on the need for explicit discharge criteria was mapped to the longitudinal monitoring and discharge phase.

This structured mapping approach ensured that the final pathway model directly reflects expert consensus rather than post hoc interpretative synthesis, thereby strengthening the conceptual validity of the proposed care itinerary. Table 3 summarizes the mapping of the Delphi items.

In cases where multiple items reached consensus within the same domain, selection for pathway integration was based on (i) strength of consensus (IQR), (ii) consistency across rounds, and (iii) clinical interpretability within a minimal, operational dataset.

Core elements of the pathway were primarily derived from items with strong consensus, whereas components with weaker agreement were incorporated as contextual or supportive elements when clinically relevant.

**Figure 4:**
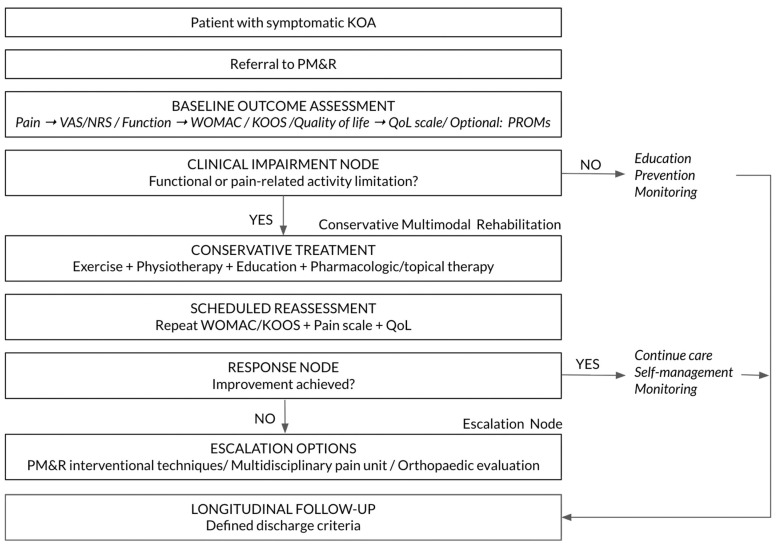
PM&R-Led Care Pathway for Knee Osteoarthritis (KOA): Baseline Outcomes, Impairment Stratification, Multimodal Conservative Rehabilitation, and Escalation with Longitudinal Follow-Up.

Figure 4 shows the algorithm that summarizes the consensus-derived clinical itinerary for patients with symptomatic KOA managed within PM&R.

### 3.4. Stability of Responses Across Rounds

Although the questionnaire remained unchanged between rounds, several items showed a reduction in IQR and an increase in central tendency, indicating convergence of expert opinion. This supports interpreting the second round as a validation and consolidation step rather than a new Delphi iteration.

## 4. Discussion

This Real-Time Delphi consensus among SERMEF PM&R physicians proposes a structured, function-oriented care itinerary for patients with KOA managed within rehabilitation services. The outputs converge on a longitudinal model centred on standardized outcome assessment, multimodal conservative care as the foundation of management, escalation guided by reassessment, and explicit discharge criteria—elements that are consistent with contemporary international guidance emphasizing exercise, education/self-management support, and person-centred care [2,9,10].

### 4.1. Function-First Framing and Measurement Strategy

A central implication of the consensus is the prioritization of function as the organizing principle of KOA rehabilitation. The strong endorsement of WOMAC, pain intensity scales, and quality-of-life measures supports a minimum dataset that captures symptom severity, disability, and broader impact. This is aligned with guideline syntheses showing that higher-quality OA recommendations consistently foreground exercise, education, and patient-centred outcomes, and with updated EULAR non-pharmacological core recommendations for hip/knee osteoarthritis [2,10,11].

The more heterogeneous endorsement of broader PROMs and subjective global ratings is plausibly explained by implementation barriers in routine care (time, workflow integration, digital infrastructure, and standardization). Recent evidence syntheses and implementation studies highlight that PROM initiatives can improve care, but successful uptake depends strongly on design and implementation strategies, including co-design with patients and clinicians [12,13].

From a pathway perspective, anchoring decisions to repeated measurement is also clinically meaningful because clinically important change thresholds are heterogeneous across instruments and contexts; contemporary syntheses provide estimates of minimal important change/difference for commonly used KOA outcome tools, supporting interpretability of longitudinal monitoring [14].

Although WOMAC was the most consistently supported functional scale, alternative instruments such as KOOS are widely used and may be equally appropriate depending on the clinical context.

### 4.2. Conservative Multimodal Care as the Backbone

The pathway structure derived from the Delphi aligns with the evidence base positioning exercise therapy and rehabilitation interventions as first-line management in KOA, noting persistent underutilization despite strong evidence [2,15,16].

The panel’s preference for integration over rigid step ordering is also coherent with real-world KOA care, where tailoring is required based on clinical phenotype, comorbidities, symptom drivers, and functional goals. Guideline syntheses further indicate that while core recommendations are consistent, variability persists in adjunctive options and in practical applicability/implementation guidance—an issue that may contribute to heterogeneity for “fixed-sequence” statements [10,11].

### 4.3. Escalation and Interventional Positioning

A clinically important convergence in this Delphi is the lack of support for interventional techniques as a first-line strategy, with interventional options positioned as escalation tools following an inadequate response to conservative management. This pattern is consistent with major guidance that prioritizes conservative interventions and recommends intra-articular therapies selectively (e.g., corticosteroid injections for acute exacerbation—particularly with effusion—rather than as routine first-line treatment) [9,17,18].

This consensus supports an escalation logic based on reassessment and function rather than procedure-led pathways, while still preserving a role for interventions as part of a stepped, individualized plan.

### 4.4. Service Configuration, Referral Routes, and Multidisciplinary Models

The consensus that KOA can be appropriately managed within general rehabilitation outpatient settings, with frequent referral from primary care, aligns with pragmatic access structures in many systems and with the emphasis on early delivery of non-pharmacological core care [2,10].

The observed convergence toward multidisciplinary musculoskeletal pain-unit models after Round 2 is consistent with emerging literature that argues for integrated, interprofessional care pathways in KOA, even though KOA-specific outcome evidence for collaboration models remains comparatively limited [19].

### 4.5. Discharge Criteria and Longitudinal Follow-Up as Quality Levers

The strong consensus on explicit discharge criteria is particularly actionable. KOA is a chronic, fluctuating condition; structured discharge criteria can standardize transitions from specialist rehabilitation to supported self-management and community/primary care follow-up, while preserving the capacity to re-enter specialist care when function deteriorates. This emphasis is coherent with guideline calls for sustained self-management strategies and ongoing exercise engagement as core elements of OA management [9,16,17].

### 4.6. Implications for Implementation

Taken together, the consensus itinerary provides a pragmatic scaffold for reducing unwarranted variability in KOA rehabilitation practice. Near-term implementation priorities supported by both consensus and contemporary guidance include: (i) embedding a minimum dataset (pain, WOMAC/function, QoL) at baseline and follow-up; (ii) ensuring consistent access to multimodal conservative care (exercise-based therapy, education/self-management support, and indicated adjunct therapies); (iii) defining escalation triggers based on reassessment; and (iv) standardizing discharge criteria and transition plans [2,9,12].

### 4.7. Strengths and Limitations

Several methodological considerations should be acknowledged. The second Delphi round incorporated additional participants recruited during a national congress, which differs from traditional fixed-panel Delphi designs. However, this strategy is consistent with real-time Delphi methodologies aimed at increasing representativeness and validating consensus across a broader expert community. The consistent eligibility criteria, identical questionnaire structure, and comparable participant characteristics between rounds support the robustness of the consensus findings.

Strengths include the Real-Time Delphi approach and the two-round structure (asynchronous followed by in-person), which plausibly promoted convergence for items with initial dispersion. Limitations include the exclusive participation of SERMEF PM&R physicians, which may have shaped the consensus toward a rehabilitation-centred perspective, particularly in the prioritization of conservative management and functional outcomes. While this focus aligns with the coordinating role of PM&R in KOA care, it may underrepresent perspectives from other key stakeholders such as primary care, rheumatology, orthopedics, physiotherapy, nursing, and patients. Therefore, the results should be interpreted as a specialty-informed consensus rather than a fully multidisciplinary guideline. Additionally, Delphi agreement does not directly establish effectiveness, highlighting the need for prospective implementation evaluation.

The absence of item modification between rounds reflects the real-time Delphi design, in which consensus refinement occurs through exposure to aggregated responses rather than questionnaire restructuring.

Items that did not reach a strong consensus in Round 1 but were retained in the final pathway (e.g., WOMAC, pain scales) were incorporated based on their improved agreement in Round 2 and their role as widely used reference measures in clinical practice.

Therefore, observed differences between rounds should be interpreted primarily as reflecting differences in interaction format (asynchronous vs. synchronous) and controlled expansion of the panel, rather than as a source of systematic bias.

### 4.8. Future Directions

Next steps should include multi-stakeholder co-design (including patients) and implementation studies assessing feasibility, fidelity, functional outcomes, patient-reported outcomes, and resource use. PROM implementation evidence indicates that impact depends on workflow integration and stakeholder engagement, supporting a structured implementation plan alongside the pathway [12,13].

## 5. Conclusions

This Real-Time Delphi study among SERMEF Physical Medicine and Rehabilitation (PM&R) physicians defines an implementation-oriented, function-centred care itinerary for patients with knee osteoarthritis managed within rehabilitation services. Expert consensus converged on a longitudinal pathway structured around five linked phases: (1) entry and referral to PM&R, typically from primary care; (2) standardized baseline assessment using a minimum dataset that includes pain intensity (VAS/NRS), function (WOMAC), and quality of life; (3) multimodal conservative rehabilitation as the foundation of management (exercise-based therapy/physiotherapy, education and self-management support, and indicated oral/topical therapies); (4) escalation guided by scheduled reassessment, with interventional PM&R techniques, multidisciplinary musculoskeletal pain-unit management, or orthopedic evaluation reserved for patients with persistent pain and/or functional limitation despite adequate conservative care; and (5) longitudinal follow-up with explicit, predefined discharge criteria supported by repeated outcome measurement.

Overall, the resulting model provides a pragmatic scaffold to reduce unwarranted variability in KOA rehabilitation and to operationalize a stepped, outcomes-driven approach centred on functioning. Future work should focus on multi-stakeholder co-design (including patients and other specialties) and prospective implementation studies to evaluate feasibility, fidelity, patient-reported outcomes, functional impact, and resource use when applying this itinerary in routine care.

These findings should be interpreted within the context of a specialty-specific consensus and may not be directly generalizable to other healthcare systems or multidisciplinary settings.

## Figures and Tables

**Table 1:** Demographic and professional characteristics of SERMEF PM&R physicians participating in both Delphi rounds.

Variable	Category	n	%
Sample	Complete respondents	110	100
Gender			
	Female	71	64.0
	Male	39	35.1
Age group			
	20–29	13	12.6
	30–39	24	21.6
	40–49	26	23.4
	50–59	31	27.9
	60–69	15	13.5
	≥70	1	0.9
Work setting			
	Tertiary/referral hospital	57	51.4
	Secondary hospital (~400 beds)	30	27.0
	General hospital (~200 beds)	9	8.1
	District hospital (~100 beds)	9	8.1
	Primary care	2	2.7
	Other	3	2.7
Experience in musculoskeletal Rehabilitation. Medicine			
	<5 years	7	7.2
	5–10 years	19	17.1
	10–20 years	38	34.2
	>20 years	46	41.4
Experience with interventional techniques for musculoskeletal pain management			
	<5 years	13	12.6
	5–10 years	27	24.3
	10–20 years	43	38.7
	>20 years	27	24.3
Weekly number of patients with knee OA evaluated per physician			
	<20	15	14.4
	20–50	46	41.4
	>50	49	44.1
Research involvement measured in publications during last 5 years			
	None	35	32.4
	<5 studies	53	47.7
	5–30 studies	15	13.5
	>30 studies	7	6.3
Where are your patients with knee osteoarthritis generally managed?			
	General rehabilitation clinic	88	80.2
	Multidisciplinary MSK pain unit	16	14.4
	Dedicated OA clinic	1	0.9
	Interventional clinic	1	0.9
	Other	4	3.6

**Table 2:** Item-level agreement and consensus metrics across the two Delphi rounds.

(a) Item-Level Agreement and Consensus Metrics Across the Two Delphi Rounds (R1 vs. R2): Pathways.										
**#**	Item (Full Text)	Mean		Δ	Median		SD		IQR	
		R1	R2	Mean	R1	R2	R1	R2	R1	R2
Q37	Ideally, defined discharge criteria should exist.	5.20	5.43	0.23	5	5	0.72	0.63	1	1
Q33	Ideally, outcome measures should include a WOMAC knee functional scale.	4.88	5.15	0.27	5	5	1.02	0.63	2	1
Q34	Ideally, outcome measures should include a quality of life scale.	4.53	4.97	0.44	5	5	0.85	0.74	1	0
Q32	Ideally, outcome measures should include a pain scale (VAS/NRS).	4.75	4.96	0.21	5	5	1.06	0.83	2	1
Q28	In knee osteoarthritis, the therapeutic sequence should include physical therapy in combination with oral and/or topical therapy and/or interventional techniques.	4.53	4.91	0.38	5	5	0.93	0.79	1	0
Q27	In knee osteoarthritis, the therapeutic sequence should be topical therapy, oral therapy, physical therapy, and interventional techniques.	4.63	4.89	0.26	5	5	1.08	0.99	1	2
Q24	Ideally, patients with knee osteoarthritis should be seen in a general rehabilitation outpatient clinic.	4.35	4.72	0.37	5	5	1.19	1.04	1	1
Q21	Ideally, patients with knee osteoarthritis should be referred from primary care or other physicians.	4.61	4.71	0.10	5	5	1.14	1.00	1	1
Q23	Ideally, patients with knee osteoarthritis should be managed in a multidisciplinary musculoskeletal pain unit.	4.43	4.51	0.08	5	5	1.47	1.17	3	1
Q25	Ideally, patients with knee osteoarthritis should be seen in a dedicated osteoarthritis clinic.	3.95	3.81	0.14	4	4	1.18	1.21	2	2
Q36	Ideally, outcome measures should include PROMs (Patient-Reported Outcome Measures).	4.05	3.80	0.25	4	4	1.11	1.24	2	2
Q35	Ideally, outcome measures should include subjective assessment scales (patient and clinician).	3.85	3.66	0.19	4	4	1.12	1.08	2	2
Q22	Ideally, patients with knee osteoarthritis should be referred from rheumatology.	2.83	3.04	0.21	3	3	0.83	1.12	1	2
Q31	Ideally, outcome measures should include a descriptive scale (same/worse/better).	2.93	2.85	0.08	3	3	0.92	0.97	1	1
Q20	Ideally, patients with knee osteoarthritis should be referred from orthopaedics (COT).	2.15	2.38	0.23	2	2	0.82	0.89	1	1
Q26	Ideally, patients with knee osteoarthritis should be seen in a dedicated interventional clinic.	2.83	2.29	0.54	3	2	0.98	0.89	2	1
Q30	Interventional techniques should be used as first-line treatment combined with physical therapy.	2.08	1.82	0.26	2	2	0.73	0.83	0	1
(**b**) **Item-Level Agreement and Consensus Metrics Across the Two Delphi Rounds (R1 vs. R2): Treatments.**										
**#**	**Item (Full Text)**	**Mean**		**Δ**	**Median**		**SD**		**IQR**	
		**R1**	**R2**	**Mean**	**R1**	**R2**	**R1**	**R2**	**R1**	**R2**
Q15	Ideally, treatment should include therapeutic exercise	5.52	5.71	0.19	6	6	0.71	0.52	0	0
Q14	Ideally, treatment should include weight control	5.41	5.65	0.24	6	6	0.76	0.60	0	0
Q9	Ideally, treatment should include oral strong opioids	2.32	2.05	0.27	2	2	0.94	0.81	1	1
Q18	Ideally, treatment should include orthoses	2.56	2.40	0.16	2	2	1.01	0.95	1	1
Q17	Ideally, treatment should include occupational therapy	2.48	2.36	0.12	2	2	0.98	0.88	1	1
Q8	Ideally, treatment should include oral tramadol	3.21	3.10	0.11	3	3	1.02	0.93	1	1
Q19	Ideally, treatment should include heat or cold therapy	3.98	4.07	0.09	4	4	1.07	1.00	2	2
Q7	Ideally, treatment should include oral NSAIDs	4.02	4.18	0.16	4	4	1.12	1.05	2	2
Q13	Ideally, treatment should include topical capsaicin	3.22	3.15	0.07	3	3	1.03	1.02	2	2
Q12	Ideally, treatment should include topical NSAIDs	4.01	3.86	0.15	4	4	1.08	1.16	2	2
Q11	Ideally, treatment should include chondroitin or glucosamine	3.25	3.12	0.13	3	3	1.15	1.18	2	2
Q10	Ideally, treatment should include duloxetine	3.08	3.01	0.07	3	3	1.06	1.12	2	2
Q6	Ideally, treatment should include classical analgesic electrotherapy	3.11	3.02	0.09	3	3	1.09	1.15	2	2
Q16	Ideally, treatment should include physiotherapy	3.04	2.92	0.12	3	3	1.14	1.19	2	2
(**c**) **Item-Level Agreement and Consensus Metrics Across the Two Delphi Rounds (R1 vs. R2): Referral Criteria.**										
**#**	**Item (Full Text)**	**Mean**		**Δ**	**Median**		**SD**		**IQR**	
		**R1**	**R2**	**Mean**	**R1**	**R2**	**R1**	**R2**	**R1**	**R2**
Q1	The ideal management of KOA patients should include early evaluation by PM&R specialists	5.34	5.58	0.24	6	6	0.81	0.63	1	0
Q2	KOA management should prioritise functional recovery as a primary clinical objective	5.46	5.69	0.23	6	6	0.77	0.58	1	0
Q3	KOA patient care should be coordinated through multidisciplinary management	5.12	5.41	0.29	5	6	0.96	0.76	1	1
Q4	PM&R services should play a central coordinating role in KOA care pathways	4.98	5.21	0.23	5	5	1.01	0.88	2	1
Q5	KOA clinical management should include structured clinical pathway protocols	4.86	5.03	0.17	5	5	1.08	0.94	2	2

**Table 3:** Mapping of Delphi items to the operational rehabilitation pathway model.

Pathway Phase	Clinical Purpose	Delphi Items Supporting the Phase
Phase 1. Entry and Referral to PM&R	Defines how patients access rehabilitation care and where initial evaluation occurs	Q21 Referral from primary care or other physicians Q24 Evaluation in general rehabilitation outpatient clinic Q20 Possible referral from orthopedics Q22 Possible referral from rheumatology
Phase 2. Functional and Outcome Assessment	Establishes baseline clinical status and enables follow-up comparison using validated outcome measures	Q32 Pain scale (VAS/NRS) Q33 WOMAC functional scale Q34 Quality of life scale Q31 Descriptive global status scale Q35 Subjective clinical and patient assessment Q36 PROMs
Phase 3. Conservative Multimodal Treatment	Defines first-line therapeutic management combining rehabilitation and supportive therapies	Q28 Multimodal treatment combining physical therapy and pharmacological/topical therapy Q27 Sequential organization of treatment components
Phase 4. Escalation and Advanced Therapeutic Options	Defines when and how advanced treatments or specialist referrals are considered	Q30 Interventional techniques should not be first-line treatment Q26 Use of interventional techniques when escalation is required Q23 Possible management in multidisciplinary MSK pain unit Q25 Dedicated OA clinics as specialized care setting
Phase 5. Longitudinal Monitoring and Discharge	Defines follow-up strategy and criteria for completing rehabilitation care	Q37 Defined discharge criteria

## Data Availability

The data presented in this study are available on request from the corresponding author due to privacy restrictions.

## Abbreviations

The following abbreviations are used in this manuscript:

KOA	Knee Osteoarthritis
PM&R	Physical Medicine and Rehabilitation
MSK	Musculoskeletal
